# Interest in healthy living outweighs presumed cultural norms for obesity for Ghanaian women

**DOI:** 10.1186/1477-7525-4-44

**Published:** 2006-07-20

**Authors:** Rosemary B Duda, Naana Afua Jumah, Allan G Hill, Joseph Seffah, Richard Biritwum

**Affiliations:** 1 Department of Surgery, Beth Israel Deaconess Medical Center, RW871, 330 Brookline Ave, Boston, MA 02215, USA; 2 Harvard Medical School, Boston, MA 02215, USA; 3 Department of Population and International Health, Harvard School of Public Health, Huntington Ave, Boston, MA 02115, USA; 4 Department of Obstetrics and Gynecology, Korle Bu Teaching Hospital, University of Ghana, Accra, Ghana; 5 Department of Community Medicine, Korle Bu Teaching Hospital, University of Ghana, Accra, Ghana

## Abstract

**Background:**

Cultural norms indicate that obesity reflects increased wealth and prosperity. Yet obesity is linked to serious medical illnesses. The purpose of this study was to determine if Ghanaian women would change their body image if it meant a healthier life.

**Methods:**

A questionnaire was administered to 305 Ghanaian women waiting for clinic appointments at Korle Bu Teaching Hospital, Accra Ghana. This survey included questions on current health, selection of figural stimuli, decision making on health and social determinants and 5 questions on self-perception of health from SF-36. Anthropometric measures were taken and body mass index calculated. Women were also provided with health related information at the conclusion of the interview.

**Results:**

The majority of all women surveyed would reduce their current body image if it meant that they would have an overall healthier life and reduce the risks of obesity-linked illnesses and complications. Currently obese women were significantly more likely than non-obese women to reduce their body image to reduce the risk of hypertension (OR 2.03 [1.64 – 2.51],<0.001); cardiovascular accident (OR 1.96 [1.61 – 2.38],<0.001); diabetes (OR 2.00 [1.63 – 2.44],<0.001); myocardial infarction (OR 2.27 [1.80 – 2.86],<0.001); if requested by a spouse(OR 2.64 [1.98 – 3.52],<0.001); and to improve overall health (OR 1.95 [1.60 – 2.37], <0.001). There was no association with current body image and responses to SF-36. The decision to select a new body image was not influenced by education, income, marital status or parity. Age 50 years old and less was significantly associated with the body image size reduction to reduce the risk of hypertension, diabetes, and a cardiovascular accident.

**Conclusion:**

The Ghanaian women interviewed in this study are interested in living a healthy life and are willing to reduce their body size to reduce the risk of obesity-linked illnesses. The target group for any interventional studies and measures to reduce obesity appears to be women age 50 and younger.

## Background

It is estimated that over 115 million people suffer from obesity related health conditions in the developing nations [1-3]. Obesity is a marker or risk factor for several illnesses, including hypertension, type 2 diabetes and cardiovascular disease [4]. An increase in body weight with an increase in age was an uncommon occurrence in Sub Saharan African populations just over a little more than a generation ago [5]. However, recent studies have shown that particularly in the urban environment the prevalence of obesity and obesity linked illnesses are increasing [6,7].

The etiology of obesity in all populations is complex [8]. The causes include genetics, diet, activity level and cultural norms as a sign of wealth and prosperity [9,10]. In a recent study of 305 women using culturally adapted figural stimuli, we found that Ghanaian women view their current body image (CBI) as overweight or obese [11]. However, 94.9% stated that they were aware of health risks associated with obesity and 47.8% selected the ideal body image (IBI) of a Ghanaian woman to be smaller than her own CBI. Of the 106 women who were obese by body mass index measurements, 88.2% preferred a smaller IBI in comparison to her own CBI. The majority of women also selected the figure representing morbid obesity as the least healthy and the healthiest figures were 2 that represented normal to slightly overweight women. The purpose of this study is to determine if women would alter their body image for specific health and/or social conditions.

## Methods

### Figural Stimuli – body images

The prototype of a culturally adapted Figural Stimuli for Ghanaian women was developed using a computerized body morph assessment tools (Adobe Photoshop and Abrosoft Fanta Morph3) [12-15]. A hand drawn figure representative of a Ghanaian woman was drawn from a combination of photographs, then scanned and morphed to include select body images that included a range of shapes to represent very thin to morbidly obese. The images were printed in color on a single placard for use in the study [11].

### Survey

A verbally administered survey was conducted that included queries on age, area of residence, ethnicity, marital status, parity, income and education level, a previous history of intentional weight gain or loss, diet and exercise habits, family or peer pressure to change their weight as an adult or child and interest in participating in a trial to reduce weight and promote healthy living [11]. The women reviewed the placard and selected her CBI and her choice of the IBI. She was also asked to decide if she would change her CBI to another body image if it meant that she would be healthier and then to select that new image. Medical conditions that are linked to obesity and two medical conditions not linked to obesity as well as one social determinant were used individually to assess the conditions that may cause the participant to change her CBI. The survey also included food access questions and questions from the Short Form 36 – a standardized self assessment of health [16].

### Anthropometric measurements

Anthropometric measurements were obtained with the women wearing lightweight street clothes without shoes. Weight was measured on a calibrated Salter scale to the nearest 0.1 kilogram (kg). Height was measured to the nearest 0.5 cm with the women standing upright with the head in the Frankfurt position [17]. Body mass index (BMI) was calculated as weight (kg) divided by height squared (meter^2^). Body mass index (BMI) was defined as BMI ≤ 18.5 underweight; BMI 18.5 – 24.9 normal weight; BMI 25.0 – 29.9 overweight; and ≥ 30.0 obese. Morbid obesity is defined as BMI ≥ 40.0 [18]. Unless specifically stated, the obese weight category refers to all those with a BMI ≥ 30.0 kg/m^2^. Waist circumference was measured in centimeters (cm) at the mid-point between the lower ribs and the iliac crest. Hip measurements were taken at the maximal circumference of the buttocks. The waist to hip ratio (WHR) was calculated from the waist and hip measurements. A WHR > 0.8 has been associated with increased risks for type 2 diabetes, coronary artery disease and hypertension [19].

At the end of the interview, the women were provided with a written copy of their measurements, the calculated Body Mass Index and an interpretation. They were also provided with a pamphlet on diet, exercise and healthy living. The length of interview ranged from 10 minutes to one hour, providing each woman with sufficient time to complete the questionnaire.

### Eligibility

All of the women who were attending the gynecologic clinic or the radiology clinic at Korle Bu Teaching Hospital, Accra, Ghana were asked to participate in the study. The women, who were otherwise waiting for the clinics to start, were required to sign an informed consent, be at least age 18 years or older, not be pregnant or breast feeding and be able to communicate with the interviewers. The interviews for the most part were conducted in English, the official language in Ghana. Occasionally the head nurse (matron) of the clinic would assist with terms if not understood by the participant.

### Statistical analysis

The data was coded and entered into SPSS version 13 for Windows (SPSS, Inc., Chicago, IL.). Analysis included frequency distributions, a dissatisfaction score (CBI-IBI), and binary logistic regression analysis. A p value of < 0.05 was considered significant. The strength of association is expressed as the Odds Ratio with a 95% confidence interval.

### Institutional reviews

The study was approved by the Committee for Clinical Investigations, Beth Israel Deaconess Medical Center; Institutional Review board, Harvard Medical School; Human Subjects Committee, Harvard School of Public Health; and the Institutional Review Board, Noguchi Memorial Institute of Medical Research, University of Ghana.

## Results

### Demographic characteristics

A total of 305 women completed the study, conducted between July and August 2005. The mean age was 35.9 years (range 19 to 74 years) with 71.1% being married, 48.2% were nulliparous, 37.1% multiparous, 94.4% receiving some level of formal education and 15.2% had no regular monthly income. There was no woman in this study who was unfamiliar with the illnesses of hypertension, diabetes, cerebral vascular accident, myocardial infarction, or malaria when asked about these conditions in local lay terms.

### Clinical characteristics

Anthropometric measurements were available for 305 women. Based on the BMI category, 1.3% (4) of the women in this study were underweight, 33.1% (101) were normal weight, 30.8% (94) were overweight and 34.8% (106) were obese. Of the 127 women in the obese BMI category, 10 met the criteria for morbid obesity. They represent 3.3% of the total women. The WHR of 172 women (56.5%) was in the obese range, indicating an even greater percentage of obesity and higher risk of obesity-linked illnesses by this alternate measure. The assessment of the participants' overall general health revealed that 18.6% were previously diagnosed as hypertensive, 2.3% were diabetics, 1.3% had suffered a previous myocardial infarction, 1.0% had suffered a previous cerebral vascular accident. 14% of the women interviewed states that they were diagnosed with obesity by a physician, most of whom offered that they were told to lose weight, and 79.5% reported at least one previous episode of malaria.

### Health and social determinants affecting change of CBI

The length of time to administer the questionnaire ranged from 10 minutes to one hour. Each woman was allowed sufficient time for her to comfortably answer the questions. Two hundred and ninety-nine women answered the health determinant questions, the women who did not had been summoned for their medical appointment before the survey was completed.

A series of health conditions and social situations were used to determine if the women would change their CBI if there was a link between the CBI and the determinant. The women were asked: 1) "If you were told that your current figure (CBI) was associated with an increased chance of developing hypertension, stroke, heart attack, diabetes, poor vision, malaria, would you change it?", and 2) "Would you change your figure if your husband or significant other asked you to do so?". If the response was "yes", the woman was asked to select the new body image (NBI) model. The scores were calculated by subtracting the NBI from the CBI, with a positive value indicating the NBI to be smaller than the CBI and a negative value indicates that the NBI is larger than the CBI.

Table 1 shows the number and percent of women who selected a NBI and the average change for each health and social determinant. The majority of women selected a NBI for all health determinants evaluated that are linked to obesity. Over one-half of the women recognized that malaria and poor vision are not linked to obesity and did not select a NBI. A surprising 63.4% of women stated that they would change their body size if requested by their husband – to an average change of 2 sizes smaller than their CBI. Only 7.7% stated that the change would be to a larger size.

**Table 1:** Health and social determinants affecting change in CBI

**Determinant**	**Frequency of Change to a New Body Image *(n = 299 total)***		**Average Change in Figures**	
	**n**	**%**	**Mean**	**sd**
Hypertension	224	74.9	2.0	1.9
Stroke	224	75.9	2.1	1.9
Diabetes	218	72.9	2.4	2.0
Heart Attack	221	73.9	2.3	2.1
Poor Vision	169	56.5	2.3	2.1
Malaria	184	61.5	2.4	2.1
Spouse/SO*	201	73.4	2.0	2.1
Improve overall health	226	75.6	2.0	2.1

* 25 had no spouse or significant other

Table 2 shows the range and degree of change in NBI for each determinant. Greater than 90% of the women who would change their figure for health or social circumstances selected a smaller model as her new figure for each determinant For each obesity-linked and both non-linked health conditions and the social condition determinants, the majority of the NBIs selected were from 1 to 4 sizes smaller than the CBI.

**Table 2:** Range and Degree of Changes in Current Body Image to a New Body Image for each Determinant

***Determinant***																	
Degree of Change*		***Hypertension***		***Stroke***		***Diabetes***		***Myocardial Infarction***		***Poor Vision***		***Malaria***		Spouse/SO**		***Improve Overall Health***	
		***n***	**%**	***n***	**%**	***n***	**%**	***n***	**%**	***n***	**%**	***n***	**%**	***n***	**%**	***n***	**%**
	-6	0.0	0.0	0	0.0	0	0.0	0.0	0.0	0	0.0	0	0.0	1	0.4	0	0.0
	-5	1	0.3	0	0.0	0	0.0	0.0	0.0	0	0.0	0	0.0	1	0.4	0	0.0
	-4	1	0.3	0	0.0	1	0.3	0.0	0.0	0	0.0	0	0.0	1	0.4	5	1.7
	-3	2	0.7	2	0.7	3	1.0	5	1.7	2	0.7	3	1.0	5	1.8	3	1.0
	-2	7	2.3	5	1.7	4	1.3	4	1.3	6	2.0	5	1.7	4	1.5	7	2.3
	-1	9	3.0	9	3.0	10	3.3	9	3.0	6	2.0	12	4.0	8	2.9	12	4.0
CBI	0	78	26.1	75	25.1	81	27.1	78	26.1	130	43.5	115	38.5	96	34.9	73	24.4
	1	78	26.1	59	19.7	56	18.7	72	24.1	45	15.1	46	15.4	52	18.9	70	23.4
	2	45	15.1	57	19.1	58	19.4	52	17.4	39	13.0	46	15.4	38	13.8	48	16.1
	3	36	12.0	31	10.4	34	11.4	33	11.0	27	9.0	27	9.0	25	9.1	34	11.4
	4	22	7.4	32	10.7	24	8.0	27	9.0	22	7.4	20	6.7	29	10.5	24	8.0
	5	10	3.3	19	6.4	14	4.7	10	3.3	9	3.0	12	4.0	9	3.3	13	4.3
	6	7	2.3	2	0.7	5	1.7	3	1.0	5	1.7	5	1.7	3	1.1	5	1.7
	7	1	0.3	3	1.0	5	1.7	4	1.3	5	1.7	6	2.0	2	0.7	3	1.0
	8	2	0.7	5	1.7	3	1.0	2	0.7	2	0.7	2	0.7	1	0.4	2	0.7
	9	0	0.0	0	0.0	1	0.3	0	0.0	1	0.3	0	0.0	.0	0.0	0	0.0
Total		299	100	299	100	299	100	299	100	299	100	299	100	274	100	299	100

*A negative value reflects the selection of a new body image that is larger than the current body image. **25 women had no spouse or significant other.

A comparison of obese to non-obese women was performed to assess if BMI influenced the decisions to select a NBI for each health and social determinant as shown in Table 3. When a NBI was selected, most women chose a figure 1 to 4 sizes smaller than her CBI. Table 4 shows the Odds Ratio and 95% confidence interval for each determinant. Obese women were significantly more likely to select a NBI in comparison to non-obese women for each determinant.

**Table 3:** Range of selected changes for a new body image for each determinant by Body Mass Index

***Determinant***																	
Degree of Change*		***Hypertension***		***Stroke***		***Diabetes***		***Myocardial Infarction***		***Poor Vision***		***Malaria***		Spouse/SO**		***Improve Overall Health***	
		Non obese	Obese	Non obese	Obese	Non obese	Obese	Non obese	Obese	Non obese	Obese	Non obese	Obese	Non obese	Obese	Non obese	Obese
		***%***	**%**	***%***	**%**	***%***	**%**	***%***	**%**	***%***	**%**	***%***	**%**	***%***	**%**	***%***	**%**
	-6	0.0	0.0	0.0	0.0	0.0	0.0	0.0	0.0	0.0	0.0	0.0	0.0	1.1	0.0	0.0	0.0
	-5	0.0	0.5	0.0	0.0	0.0	0.0	0.0	0.0	0.0	0.0	0.0	0.0	1.1	0.0	0.0	0.0
	-4	1.0	0.0	0.0	0.0	1.0	0.0	0.0	0.0	0.0	0.0	0.0	0.0	1.1	0.0	1.9	1.5
	-3	1.9	0.0	1.9	0.0	2.9	0.0	3.9	0.5	1.9	0.0	2.9	0.0	5.6	0.0	2.9	0.0
	-2	4.9	1.0	3.9	0.5	2.9	0.5	3.9	0.0	4.9	0.5	3.9	0.5	3.3	0.5	5.8	0.5
	-1	7.8	0.5	7.8	0.5	6.8	1.5	7.8	0.5	4.9	0.5	8.7	1.5	7.8	0.5	8.7	1.5
CBI	0	42.7	17.4	35.9	19.0	42.7	18.5	40.8	18.5	61.2	33.8	53.4	30.3	52.2	26.1	37.9	16.9
	1	30.1	23.6	29.1	14.9	25.2	15.4	28.2	21.5	13.6	15.9	19.4	13.3	22.2	17.4	28.2	21.0
	2	6.8	19.5	14.6	21.5	11.7	23.6	11.7	20.5	8.7	15.4	6.8	20.0	3.3	19.0	11.7	18.5
	3	2.9	16.9	3.9	13.8	5.8	14.4	2.9	15.4	2.9	12.3	1.9	12.8	0.0	13.6	1.9	16.4
	4	0.0	11.3	1.0	15.9	0.0	12.3	1.0	13.3	1.0	10.8	1.0	9.7	2.2	14.7	1.0	11.8
	5	0.0	5.1	1.9	8.7	0.0	7.2	0.0	5.1	0.0	4.6	1.0	5.6	0.0	4.9	0.0	6.7
	6	1.9	2.6	0.0	1.0	0.0	2.6	0.0	1.5	0.0	2.6	1.0	2.1	0.0	1.6	0.0	2.6
	7	0.0	0.5	0.0	1.5	0.0	2.6	0.0	2.1	0.0	2.6	0.0	3.1	0.0	1.1	0.0	1.5
	8	0.0	1.0	0.0	2.6	1.0	1.0	0.0	1.0	0.0	1.0	0.0	1.0	0.0	0.5	0.0	1.0
	9	0.0	0.0	0	0.0	0.0	0.5	0.0	0.0	1.0	0.0	0.0	0.0	0.0	0.0	0	0.0
Total n		103	195	103	195	103	195	103	195	103	195	103	195	90	184	103	195

*A negative value reflects the selection of a new body image that is larger than the current body image. ** 25 women had no spouse or significant other.

**Table 4:** Comparison of Obese Women to Non-obese Women Selecting a New Body Image for Health and Social Determinants

**Determinant**	**Odds Ratio**	**95.0% C.I.**	**p value**
Hypertension	2.03	1.64 – 2.51	<0.001
Stroke	1.96	1.61 – 2.38	<0.001
Diabetes	2.00	1.63 – 2.44	<0.001
Myocardial Infarction	2.27	1.80 – 2.86	<0.001
Poor Vision	1.87	1.51 – 2.30	<0.001
Malaria	1.96	1.59 – 2.43	<0.001
Spouse/SO	2.64	1.98 – 3.52	<0.001
Improved Overall Health	1.95	1.60 – 2.37	<0.001

The decision to select a NBI for each determinant was not influenced by increasing education, income, marital status or parity. Women age 19 to 50 years old were significantly more likely than older women to select a NBI if it would reduce the risk of hypertension (85.3% vs. 14.7%, respectively, p = 0.017), stroke (85.1% vs. 14.9%, respectively, p = 0.01), diabetes (85.1% vs. 14.9%, respectively, p = 0.012); a myocardial infarction (85.3% vs. 14.7%, respectively, p = 0.017); and poor vision (83.9% vs. 16.1%, respectively, p = 0.012).

### Short-Form 36 results – health self-assessment

Five questions from Short Form 36 were included in this survey. These questions were included to provide an assessment of the women's perception of her own health. In general, their overall health was perceived as excellent (15.8%), very good (25.2%), good (46.6%), and poor (12.4%). Compared to one year ago, they report that their health is much better (22.5%), somewhat better (22.5%), same (35.6%), somewhat worse (13.8%) and much worse (5.7%). Over the next year, the women would expect their health to much improve (70.1%), somewhat improve (14.8%), stay the same 14.8% or get somewhat worse (0.3%). Compared to her friends, the women felt that her own health was much better (36.0%), somewhat better (25.6%), same (28.3%), somewhat worse (8.7%) or much worse (1.4%). When asked to describe the statement "I expect my health to worsen over the next year", the women reported that it was mostly true (1.0%), somewhat true (0.7%), neither true nor false (0.7%), somewhat false (8.1%) and mostly false (89.6%). For each of the SF-36 questions, there was no significant difference between women who selected a NBI and those who did not for each health and social determinant.

### Food security and preparation

Less than 1% of the women stated that they often did not have enough food to eat, 58.7% had enough to eat and enough of the types of food they wanted to eat, while 27.5% had enough to eat but not always the types of food they desired. In general, either the woman (78.1%) or her female elder relative (9.4%) did the food shopping and prepared the meals (92.3%) for the family.

### Interest in future diet and exercise program

A total of 279 women (94.9%) stated that they were aware that there was health risks associated with being overweight or obese. One hundred and 84 (86%) of the 214 overweight or obese women stated that they would be willing to decrease their body weight by dietary and exercise interventions if it meant that they would lead a healthier life. 186 of these women stated that they would be interested in participating in a weight reduction clinical trial. Two hundred and nine women said that most likely their spouse or significant other would not object if they would want to lose weight.

## Discussion

Figural stimuli are an easy to administer self-report of body image [20]. The scale is highly robust, highly correlated with measured weight, a reliable predictor of obesity and has been widely used in epidemiologic investigations as an adjunct to measured or self-reported height and weight [21].

Body image assessment techniques include perceptual measures and attitudinal measures [12]. These measures assess the size perception accuracy and the subjective component of body image. Figural stimuli was the body image assessment tool chosen to evaluate in this investigation. Consideration was given to computerized morph models, but because of the research setting where electrical power is not always dependable, a more portable model was selected. When designing the Ghanaian figural stimuli, the models were created on a computerized interval scale rather than an ordinal scale and we also included models that represented the far extremes of weight from cachexia to morbidly obesity.

Younger women were significantly more likely than women age 51 years and older to change their current body image to reduce the risk of hypertension, diabetes, myocardial infarction, stroke and poor vision. This may reflect an attitude of older women that it is too late to improve their health or younger women hoping to maintain good health and are willing to make sacrifices to do so. This point should be further evaluated in future studies.

Interest in health conditions associated with obesity and improving their health was keen in this group of Ghanaian women. Most of the women were in good to excellent health, and with the exception of malaria, few had reported serious health problems. Most also reported that they had enough food and of the type they wished to eat. Many women had already made attempts to lose weight by diet and/or exercise. Use of diet medications is not yet popular in this culture. Known obesity-linked illnesses (hypertension, stroke, myocardial infarction, diabetes and two non-related medical conditions (malaria and poor eyesight) were used as a means to determine if women would change their own BMI for health related reasons. Over 75% of the women expressed an interest in changing her CBI for the improvement of health. Two-thirds of the women would also change their body size if requested by a spouse or significant other to improve their health.

The high percentage of women preferring a smaller figure for a healthier life was not anticipated based on purported cultural norms that suggest women prefer to be of a larger figure, so called "traditionally built" as a sign of wealth and prosperity and as a means to secure a husband. When the women agreed that they would change their figure if asked by their husband or significant other, the majority indicated that the new figure would be smaller than their current one. This indicates that their perception is that the spouse prefers slimmer women. This information is important in planning future health initiatives to reduce obesity, hypertension and diabetes in this population. While there may exist some resistance to lose weight because of the cultural value on weight and the impact of the husband's preference, most women would lose weight to live a healthier life.

Every attempt was made to select all women at the Gynecology and Radiology clinics who would be willing to participate. The limitations for patient selection included communication barriers in the various local languages and hence some interested women could not be interviewed. It is uncertain if this would have affected the final results of the study, but the presumption is that a greater cultural influence would have been captured if we were able to interview a broader section of the population. Hence, this study is not representative of all Ghanaian women in Accra, but rather represents a cross section of women who tend towards being more educated and comfortable conversing in English.

Because of the manner in which women were selected for the study, this is not a prevalence study for obesity. But the high percentage of women who were found to be overweight or obese is not surprising. The Women's Health Study of Accra, a representative sampling of 1300 adult women residing in Accra in 2003, found that 57.2% were either overweight or obese by anthropometric measurements [7]. The result from this present study also identified a high proportion of women who are overweight and obese.

## Conclusion

This information on ideal body size is important not only for promoting a healthy BMI for an individual woman, but also in establishing acceptable health policies for women's health in general. No longer can the excuse be made against weight reduction programs to reduce hypertension and diabetes risk that the women prefer to be of a large size. With this information as ammunition, plans can go forward to initiate diet and exercise programs to reduce the risks of obesity and obesity-linked illnesses. Particularly in resource limited countries, an adherence to a healthy lifestyle is less expensive than life long medication or complications as a result of obesity-linked illnesses. It appears that the ideal group of women to target initially are women age 50 years and younger for health improvement strategies. An educational program that explains the association between obesity and heart disease and diabetes would be of benefit to women of all age groups.

## Competing interests

The author(s) declare that they have no competing interests.

## Authors' contributions

RBD concept, supervision, interviews, data analysis, writing manuscript; NAJ contributed to study design, conducted most of the interviews, contributed to writing manuscript; JF contributed to study design, supervision of NAJ, contributed to writing manuscript, facilitated permission to conduct interviews at Korle Bu Teaching Hospital and University of Ghana IRB approval process; AH assisted with concept, design, data analysis and review of manuscript; RB contributed to study design, supervision of NAJ, contributed to writing manuscript, facilitated University of Ghana IRB approval process
